# *Ralstonia pseudosolanacearum* PhcQ Controls Quorum Sensing‐Dependent Phenotypes by Binding PhcA and Maintaining Its Protein Stability

**DOI:** 10.1111/mpp.70308

**Published:** 2026-07-01

**Authors:** Min Chen, Lili Luo, Huixin Yang, Liujie Hu, Tao Liang, Masayuki Tsuzuki, Yasufumi Hikichi, Kouhei Ohnishi, Tao Guo, Chengtao Li, Peng Li, Yong Zhang

**Affiliations:** ^1^ College of Environmental Science and Engineering Shaanxi University of Science and Technology Xian China; ^2^ College of Resources and Environment Southwest University Chongqing China; ^3^ HanHong College Southwest University Chongqing China; ^4^ Chongqing Academy of Agricultural Sciences Chongqing China; ^5^ Faculty of Agriculture and Marine Science Kochi University Nankoku Japan; ^6^ College of Life Sciences Hainan Normal University Haikou China

## Abstract

The quorum‐sensing (QS) system plays a crucial role in regulating the virulence of the *Ralstonia solanacearum* species complex (RSSC). It is controlled by the *phcBSR* operon and *phcA* genes. PhcQ may contribute to the activation of PhcA and regulates the expression of QS‐dependent genes. Here, we genetically demonstrate that PhcQ functions as a key component of the QS regulatory pathway by maintaining the protein stability of PhcA in *R. pseudosolanacearum* OE1‐1. Deletion of *phcQ* significantly increased the expression of genes encoding the type III secretion system but decreased the expression of genes for the synthesis of exopolysaccharide, consistent with that of PhcA. The *phcQ* mutants exhibited enhanced growth at early stages and retained weak virulence on tobacco plants, although they are nonvirulent on tomato plants. Using an overexpression system, we positioned PhcQ downstream of PhcB and upstream of PhcA in the QS regulatory cascade. PhcQ did not affect *phcA* transcription, while proteomic analysis and western blot revealed that PhcQ maintains PhcA protein stability, with PhcA protein levels reduced to approximately 41% in *phcQ* mutants. Further structural analysis revealed a disordered tail within the C‐terminus of PhcA that is dispensable for PhcA function yet critical for protein stability. Using bimolecular fluorescence complementation and GST pull‐down assay, we demonstrated that PhcQ binds directly to PhcA both in vitro and *in planta*, and deletion of this C‐terminus tail impaired their binding affinity. These findings reveal a novel regulatory mechanism where PhcQ binds to PhcA and maintains its protein stability to ensure proper QS‐dependent gene regulation in RSSC.

## Graphical abstract

PhcQ is a key intermediary stabiliser of PhcA controlling quorum sensing‐dependent phenotypes by maintaining protein stability of PhcA. It is downstream of PhcB and upstream of PhcA in the QS regulatory cascade. It binds to PhcA; deletion of a disordered tail in the PhcA C‐terminus impairs its binding affinity.

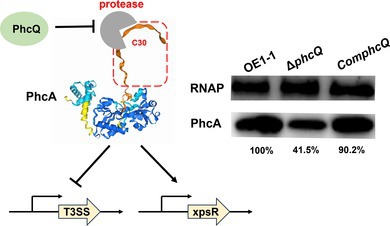

## 1 Introduction

Quorum sensing (QS) is a widely conserved cell‐to‐cell communication mechanism that plays a crucial role in regulating virulence of many pathogenic bacteria, including the *Ralstonia solanacearum* species complex (RSSC), a causative agent of bacterial wilt disease in abundant plant species worldwide (Papenfort and Bassler 2016; Kai 2023). The RSSC is composed of four phylotypes, I–IV, and is currently divided into three subgroups: *R. solanacearum* (phylotype II), *Ralstonia syzygii* (phylotype IV) and *Ralstonia pseudosolanacearum* (phylotype I and III) (Safni et al. 2014). As a soil‐borne gram‐negative bacterium, the RSSC generally infects plant hosts from the root and colonises water‐transporting xylem vessels of plants, leading to inhibition of water flow and quick plant wilting (Genin and Denny 2012; Vailleau and Genin 2023).

Many virulence determinants have been identified in RSSC, including chemotaxis and motility for initial root attachment; the type III secretion system (T3SS) and related effector proteins (T3Es) to suppress plant innate immunity and promote extensive multiplication within the host; the type II secretion system (T2SS) and secreted cell wall‐degrading enzymes (CWDEs); exopolysaccharides (EPS) synthesis and biofilm production within xylem vessels (Vailleau and Genin 2023). RSSC globally modulates these virulence determinants at various infection stages to promote host infection, and this complex regulatory network is controlled by the QS system via the key regulator PhcA, a LysR‐type transcriptional regulator, in response to cell density (Genin and Denny 2012; Kai 2023). The QS in RSSC, so‐called Phc QS, is encoded by the *phcBSRQ* operon: PhcB is a methyltransferase that catalyses formation of (*R*)‐methyl 3‐hydroxymyristate (3‐OH MAME) or (*R*)‐methyl 3‐hydroxypalmitate (3‐OH PAME), two structurally similar QS signals; PhcS and PhcR comprise a two‐component regulatory system that responds to threshold concentrations of 3‐OH MAME or 3‐OH PAME, and activate functional PhcA (Clough et al. 1997; Kai 2023). PhcA regulates expression of numerous virulence‐related genes, that is, positively regulates production of EPS and CWDEs, while it negatively regulates expression of genes for the T3SS and motility, especially at the late stage of infection within xylem vessels (Schell 2000; Hikichi et al. 2007). The Phc QS system regulates expression of about 30% of all genes in RSSC, including those related to cellular activity, primary and secondary metabolism, and pathogenicity (Perrier et al. 2018). *phcQ* is annotated as encoding a transcriptional regulator located in close proximity to the *phcBSR* genes, with only a 61‐bp gap. Transcriptome results have revealed that PhcQ positively and negatively regulates expression of 96.8% and 66.9% of QS‐dependent genes, respectively, which displays a high degree of consistency with PhcA in regulating expression of QS‐dependent genes in *R. pseudosolanacearum* OE1‐1. PhcQ might contribute to activation of PhcA and regulates expression of QS‐dependent genes (Takemura et al. 2021). It has been experimentally confirmed that PhcA directly binds to a conserved 14‐bp PhcA box in the promoters of *xpsR* (EPS), *egl* (endoglucanase) and *prhIR* (T3SS) (Huang et al. 1998; Yoshimochi, Hikichi, et al. 2009). It is worth noting that PhcA represses *prhIR* expression at high cell densities, thereby negatively regulating the downstream T3SS expression. It remains unclear how PhcQ regulates expression of these QS‐dependent genes.

In this study, we investigate the functional role of PhcQ in the regulatory cascade of the Phc QS system in RSSC and demonstrate that PhcQ acts as a crucial intermediary regulator between PhcB and PhcA, specifically by maintaining PhcA protein stability. These findings reveal a novel regulatory mechanism where PhcQ prevents the degradation of PhcA, thereby ensuring proper QS‐dependent gene regulation. It provides novel insights into complex regulatory networks controlling RSSC virulence and highlights potential targets for disease management strategies.

## 2 Results

### 2.1 PhcQ Exhibits Consistent Regulatory Behaviour as PhcA in the RSSC

**FIGURE 1:**
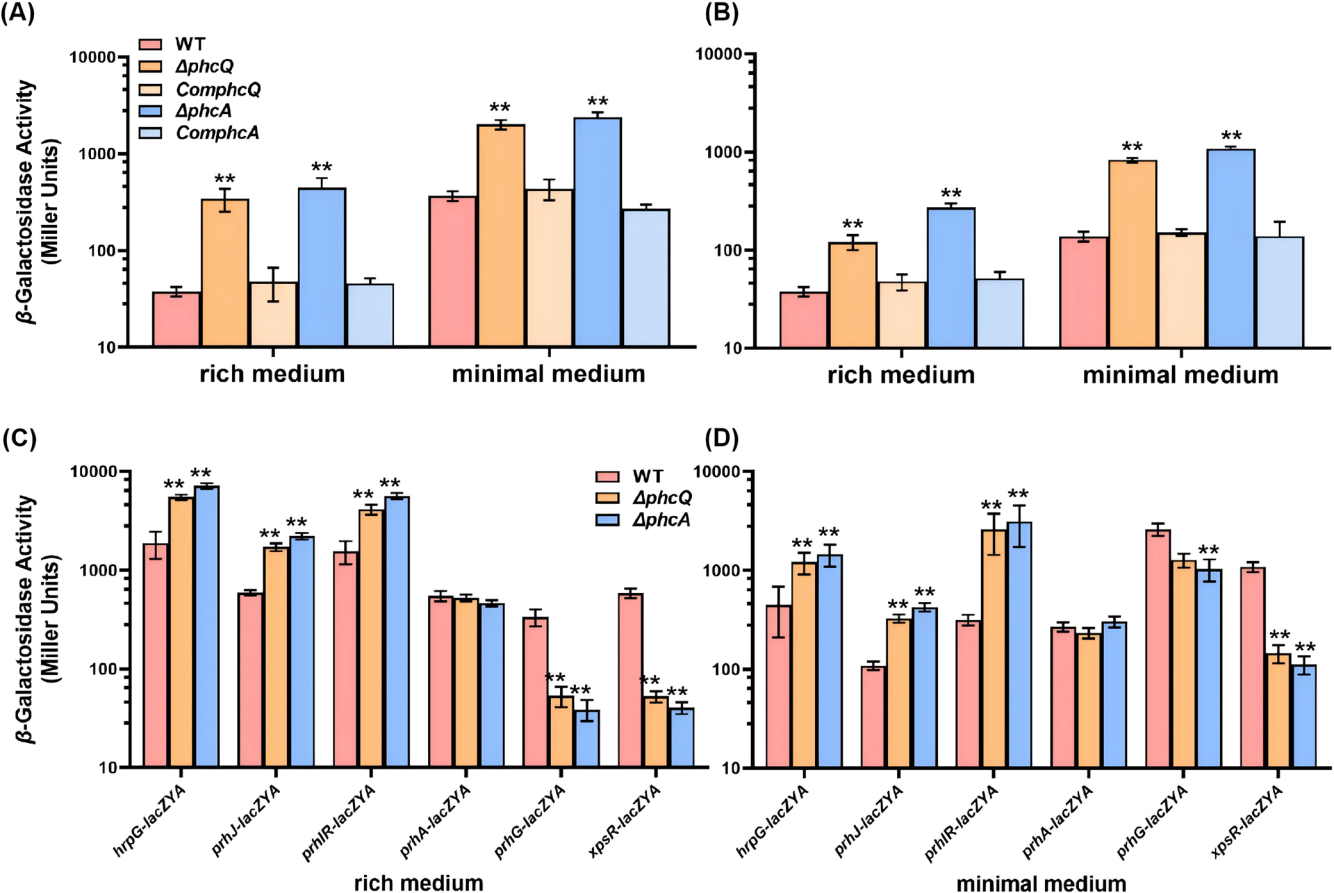
Regulatory behaviour of PhcQ and PhcA on expression of the *hrp* genes and *hrp*‐regulating genes in *Ralstonia pseudosolanacearum* OE1‐1. (A) Expression of *popA‐lacZYA* and (B) *hrpB‐lacZYA* in rich medium and minimal medium (Hoagland medium with 2% of sucrose, also used as *hrp*‐inducing medium); (C, D) expression of *hrp*‐regulating genes in rich (C) and minimal medium (D). Wild type (WT), each reporter strain of RK5050 (OE1‐1, *popA‐lacZYA*), RK5046 (*hrpB‐lacZYA*), RK5120 (*hrpG‐lacZAY*), RK5124 (*prhJ‐lacZAY*), RK5130 (*prhIR‐lacZAY*), RK5134 (*prhA‐lacZAY*), RK5212 (*prhG‐lacZAY*) and RK5138 (*xpsR‐lacZAY*). Δ*phcQ* refers to *phcQ* deletion from each reporter strain; *ComphcQ* refers to complementary *phcQ* in each *phcQ* mutant. Strains were grown in medium to about 0.1 OD_600_ and subjected to the enzymatic assay. Enzymatic activities are presented in Miller Units. Each assay was performed with three biological replicates with three replicates per trial. Mean values of all experiments were averaged with SD. Statistical significance between mutants and parent strains was assessed using a post hoc Dunnett test following ANOVA. **p < 0.01.

PhcA plays a dual role in regulating numerous genes responsible for virulence: it indirectly represses the T3SS expression by binding to the promoter of *prhIR* (Yoshimochi, Hikichi, et al. 2009). Conversely, it positively regulates EPS synthesis by binding to the promoter of *xpsR* (Huang et al. 1998). The T3SS expression is regulated by a regulatory cascade of PrhA‐PrhIR‐PrhJ‐HrpG‐HrpB. The *phcQ* gene was deleted from the following reporter strains involved in the cascade: RK5134 (*prhA‐lacZAY*), RK5130 (*prhIR‐lacZAY*), RK5124 (*prhJ‐lacZAY*), RK5120 (*hrpG‐lacZAY*), RK5046 (*hrpB‐lacZAY*), RK5050 (*popA‐lacZAY*) and RK5212 (*prhG‐lacZAY*). Deletion of *phcQ* significantly increased the expression of *prhIR*, *prhJ*, *hrpG, hrpB* and *popA* in both *hrp*‐inducing and rich media, but had no effect on *prhA* expression (Figure 1A–D). Complementation of *phcQ* decreased the expression of *popA* and *hrpB* to that of parent strains (Figure 1A,B). HrpG and PrhG are close paralogs of the two‐component system (TCS) response regulators; each positively regulates T3SS expression in parallel ways, while *prhG* expression itself is positively regulated by PhcA (Plener et al. 2010; Zhang et al. 2013). Similar to PhcA regulatory manner, *phcQ* deletion significantly decreased the expression of *prhG and xpsR* (Figure 1C,D). All these regulatory behaviours of PhcQ are fully consistent with those of PhcA in these genes.

### 2.2 PhcQ Consistently Contributes to Growth as PhcA in the RSSC

**FIGURE 2:**
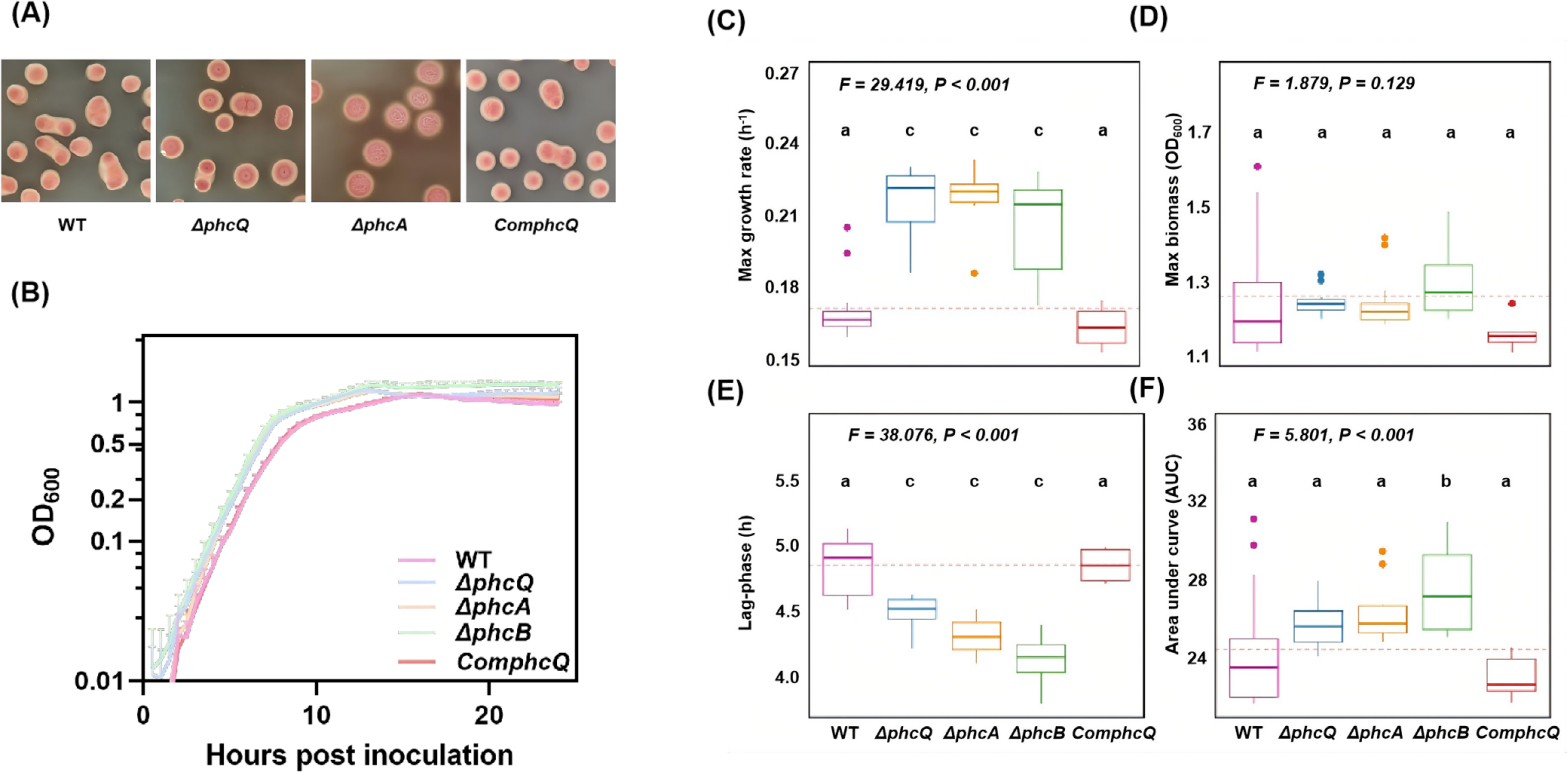
Effect of PhcQ, PhcB and PhcA on growth of *Ralstonia pseudosolanacearum* OE1‐1. (A) Morphology of single bacterial colonies on agar plates; (B) growth curves in rich medium; (C) maximum growth rate, biomass (D), lag phase (E) and area under growth curve (F). Wild type (WT, RK5050); Δ*phcQ*, RQ6289; Δ*phcA*, RQ6820. About 100 μL of cell suspension at 10^3^ cfu mL^−1^ was spread onto broth agar plates supplemented with 1% triphenyl tetrazolium chloride (TTC). Morphology of colonies was photographed after 48 h. This assay was repeated three times and representative results are presented. For growth curve monitoring, overnight cultivation of each strain was resuspended in fresh medium and adjusted to an OD_600_ of 0.01. The cell suspension was transferred into a microplate containing 200 μL, and growth curves were recorded automatically with shaking at 28°C using an automatic microbial growth curve analyser (MGC‐500; SCIENTZ). Each assay was performed with three biological replicates, including four replicates per trial. The boxes represent the interquartile range of the 25th–75th percentile of data. Lines and dots represent medians and individuals, respectively. Statistical significance across all strains was assessed using an LSD test following ANOVA. Lowercase letters above box plots represent significant differences.

The *phcA* mutants lack EPS, forming small, round, nonmucoid colonies on BGT plates (Khokhani et al. 2017). The *phcQ* mutant exhibited a similar colony morphology to *phcA* mutants but did not appear as shrivelled; *phcQ* mutant colonies were slightly smoother (Figure 2A), indicating that the EPS in *phcQ* mutants might not be completely eliminated, as it is in *phcA* mutants. Complementation of *phcQ* restored the typical mucoid colony morphology.

Mutations in PhcA enhance the environmental fitness of RSSC strains, that is, a *phcA* mutant exhibits a better growth rate than the wild‐type strain (Peyraud et al. 2016). The *phcQ* mutants also grew faster than the wild‐type strain both in rich medium (Figure 2B) and in *hrp*‐inducing medium (data not shown), but achieved an equal maximal biomass to that of the wild‐type strain (Figure 2C). Complementation of *phcQ* delayed its growth rate to that of the wild‐type strain. The maximal growth rates of *phcQ*, *phcA* and *phcB* mutants were equal (approximately 0.225 OD units/h, with no significant difference as shown in Figure 2D), but were significantly higher than that of the wild‐type strain (approximately 0.170 OD units/h, *p* < 0.001). Moreover, three mutants exhibited significantly shorter lag phases than the wild‐type strain, indicating a faster adaptation to growth (Figure 2E). Growth was also quantified as area under the growth curve. Intriguingly, the *phcQ* and *phcA* mutants displayed equal areas under the growth curve (Figure 2F), which were slightly higher than that of the wild‐type strain, but significantly less than that of *phcB* mutants (*p* < 0.01), indicating that *phcB* mutants grew fastest among these three mutants. These results confirmed that PhcQ and PhcA consistently contribute to growth in medium.

### 2.3 PhcQ Is Important for Pathogenicity and Proliferation in Tobacco Plants

**FIGURE 3:**
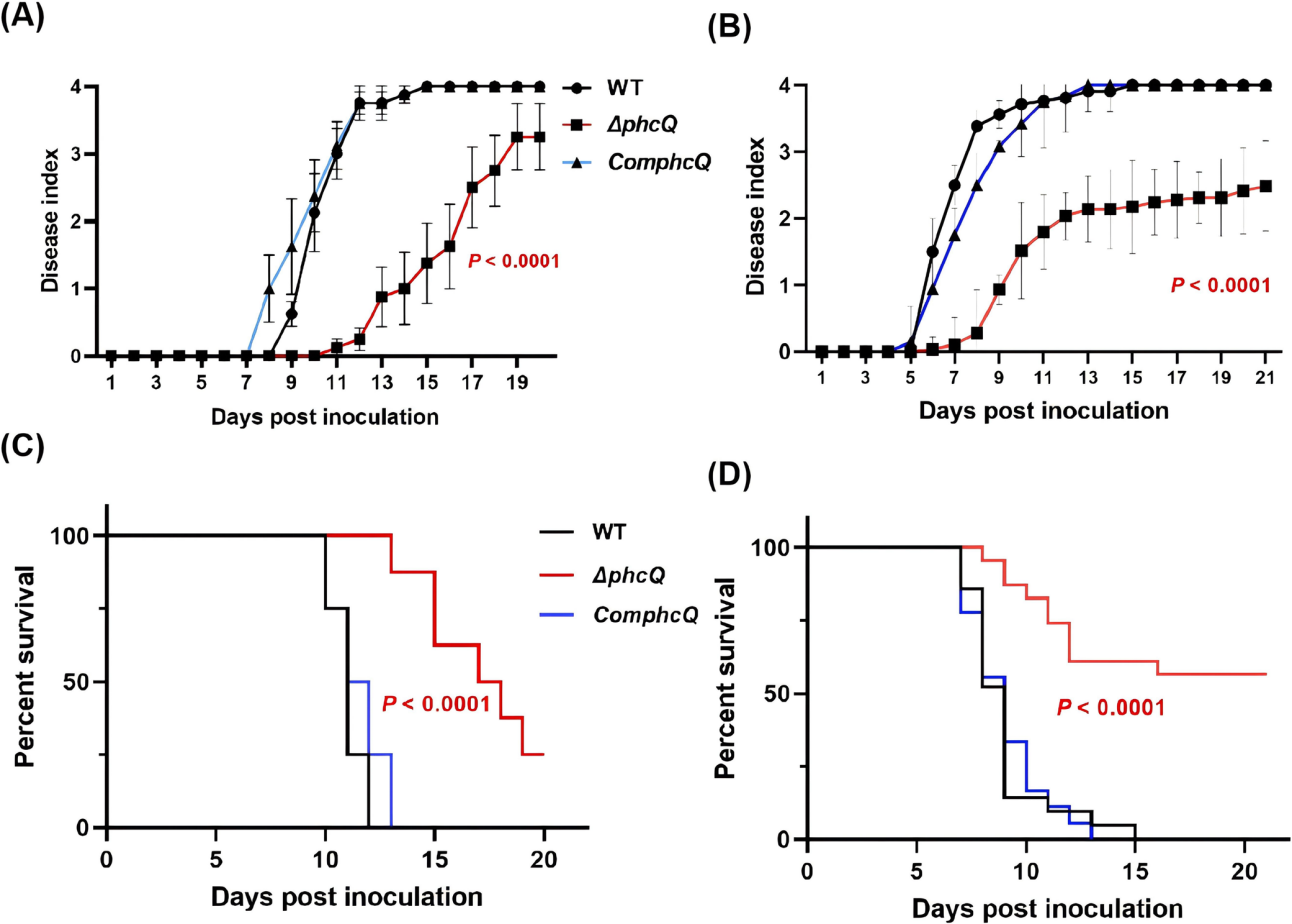
Virulence test. (A, B) Tobacco plants inoculated by soil‐soaking (A) or leaf‐infiltration (B). Wild type (WT, RK5050); Δ*phcQ*, RQ6289; *ComphcQ*, RQC1218. (C, D) Plant survival curves for A and B, respectively. For the soil‐soaking inoculation, a bacterial suspension was poured into the pot at a final cell density of 10^7^ cfu g^−1^ of soil. For the leaf infiltration, about 50 μL of bacterial suspension at 0.1 OD_600_ was infiltrated into tobacco leaves with a blunt‐end syringe. Wilt symptoms were inspected daily and scored on a disease index scale from 0 to 4 (0, no wilting; 1, 1%–25% wilting; 2, 26%–50% wilting; 3, 51%–75% wilting; 4, 76%–100% wilted or dead). Survival curves were recorded with two levels: no wilting symptoms (disease index below 3) or completely wilting (disease index equal to or higher than 3). Each assay was performed with four biological replicates, including 12 plants per trial. Mean values of all experiments were averaged with SD. Statistical significance between mutants and the wild‐type strain was assessed using a post hoc Dunnett test following ANOVA.

The *phcQ* mutants have completely lost pathogenicity on tomato plants (Takemura et al. 2021). In contrast, they retained weak virulence on tobacco plants with both inoculation methods of leaf infiltration and soil soaking, infection process of which was significantly delayed by approximately 3 to 5 days (Figure 3A–D). The *phcQ* mutants eventually wilted approximately 75% of tobacco plants with leaf‐infiltration inoculation at 21 days post‐infiltration (dpi), and wilted approximately 50% of tobacco plants with soil‐soaking inoculation at 21 dpi. Complementation of *phcQ* fully restored the impaired virulence of *phcQ* mutants to that of the wild‐type strain on tobacco plants.

**FIGURE 4:**
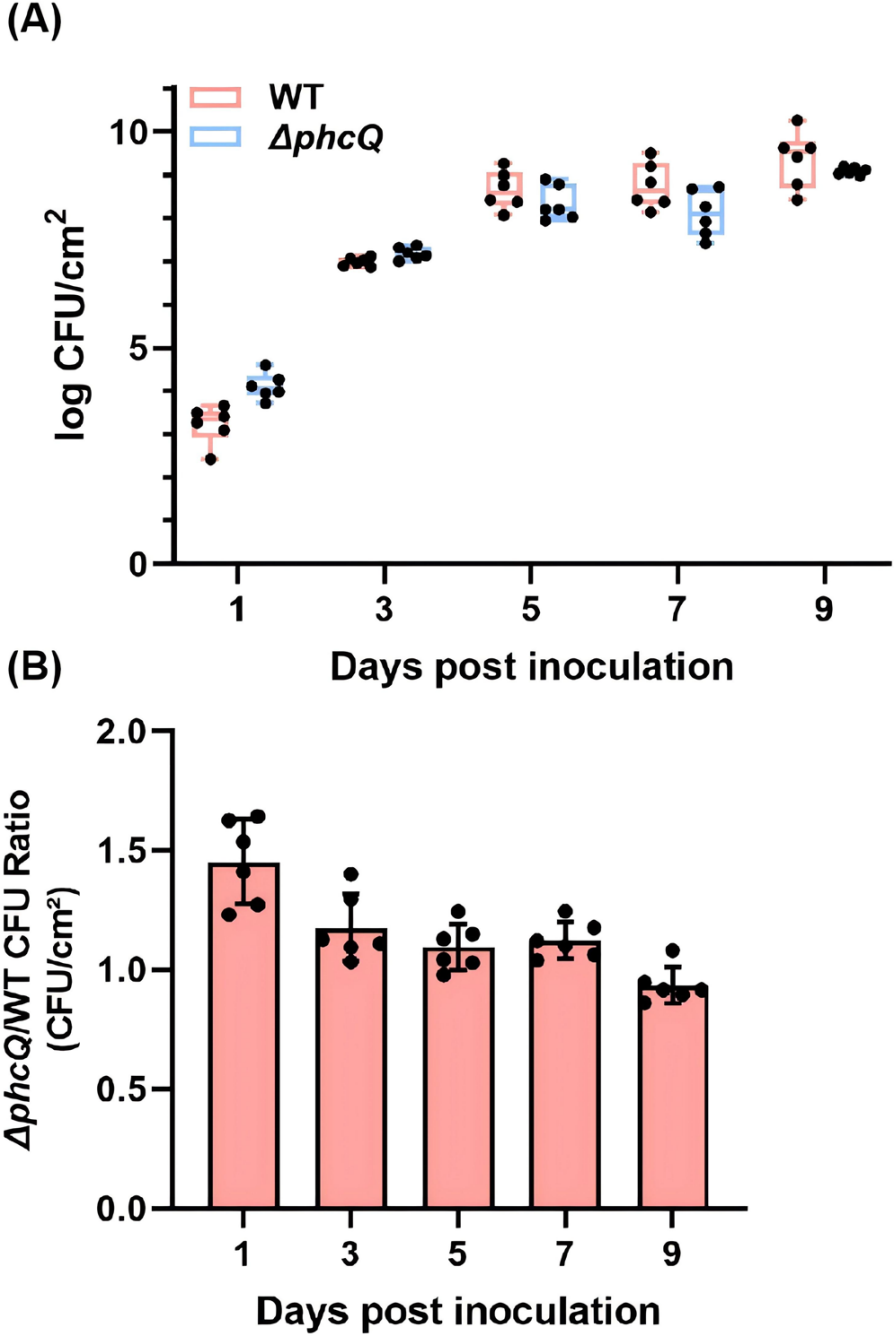
Effect of PhcQ on growth of *Ralstonia pseudosolanacearum* OE1‐1 in tobacco leaves. (A) Tobacco leaves were infiltrated with cell suspension of wild type (WT) or Δ*phcQ*. (B) Competitive fitness assay between WT (Km^r^) and Δ*phcQ* (Gm^r^). For the leaf infiltration, about 50 μL of bacterial suspension at 10^4^ cfu mL^−1^ was infiltrated into leaves with a blunt‐end syringe. Bacteria recovered from tobacco leaves were counted by dilution plating and presented in log_10_ CFU cm^−2^. For the competitive index (CI), bacterial suspension of WT (Km^r^) and Δ*phcQ* (Gm^r^) mixed at 1:1 was infiltrated into leaves. Bacteria recovered from leaves were diluted and simultaneously spread onto agar plates with Km or Gm. The ratio of Δ*phcQ* to WT was calculated. Each assay in tobacco leaves was performed with four biological replicates, including six plants per trial (*n* = 24 leaf discs at each time point). Mean values of all data were calculated into the median and horizontal lines refer to a median of 24 values at each time point. Statistical significance was assessed using a post hoc Dunnett test following ANOVA.

Proliferation inside plants is essential for pathogenicity of the RSSC (Jacobs et al. 2012; Planas‐Marquès et al. 2020). We assessed daily proliferation of the *phcQ* mutant in tobacco leaves. A bacterial suspension of the *phcQ* mutant at 10^4^ cfu/mL was infiltrated into tobacco leaves, and cell densities were quantified with the dilution plating every other day. The wild‐type strain proliferated extensively in leaves, reaching the maximum of approximately 10^9^ cfu/cm^2^ by 7 to 9 dpi. At this point, infiltrated leaves became withered and died. The *phcQ* mutant started at approximately 10^4^ cfu/cm^2^ at 1 dpi, which was significantly higher than that of the wild‐type strain (Figure 4A). It reached the maximum of approximately 10^8^ cfu/cm^2^ by 6 to 7 dpi, about one order of magnitude less than that of the wild‐type strain, although it displayed similar proliferation to the wild‐type strain from 3 to 5 dpi (Figure 4A), indicating that PhcQ is important for proliferation inside host plants, especially at an early stage.

The competition between *phcQ* mutant and the wild‐type strain was further assessed by calculating competitive index (CI) values (cell density of *phcQ* mutant versus the wild‐type strain). A bacterial suspension mixture in a 1:1 ratio of the *phcQ* mutant, marked with the Gm^r^ gene, and the wild‐type strain, marked with the Km^r^ gene, was infiltrated into tobacco leaves, and the cells of each were quantified on agar plates with corresponding antibiotics. At 1 dpi, the *phcQ* mutant outcompeted the wild‐type strain (CI = 1.5, Figure 4B), which was consistent with growth profiles of *phcQ* mutant in tobacco leaves at 1 dpi (Figure 4A). The CI values rapidly declined to about 1.0 during 3 to 9 dpi (Figure 4B), indicating that *phcQ* mutant grew similarly to the wild‐type strain in tobacco leaves, but slightly better during the initial infection process.

### 2.4 PhcQ Is Located Downstream of PhcB and Upstream of PhcA in the QS Regulatory Pathway

**FIGURE 5:**
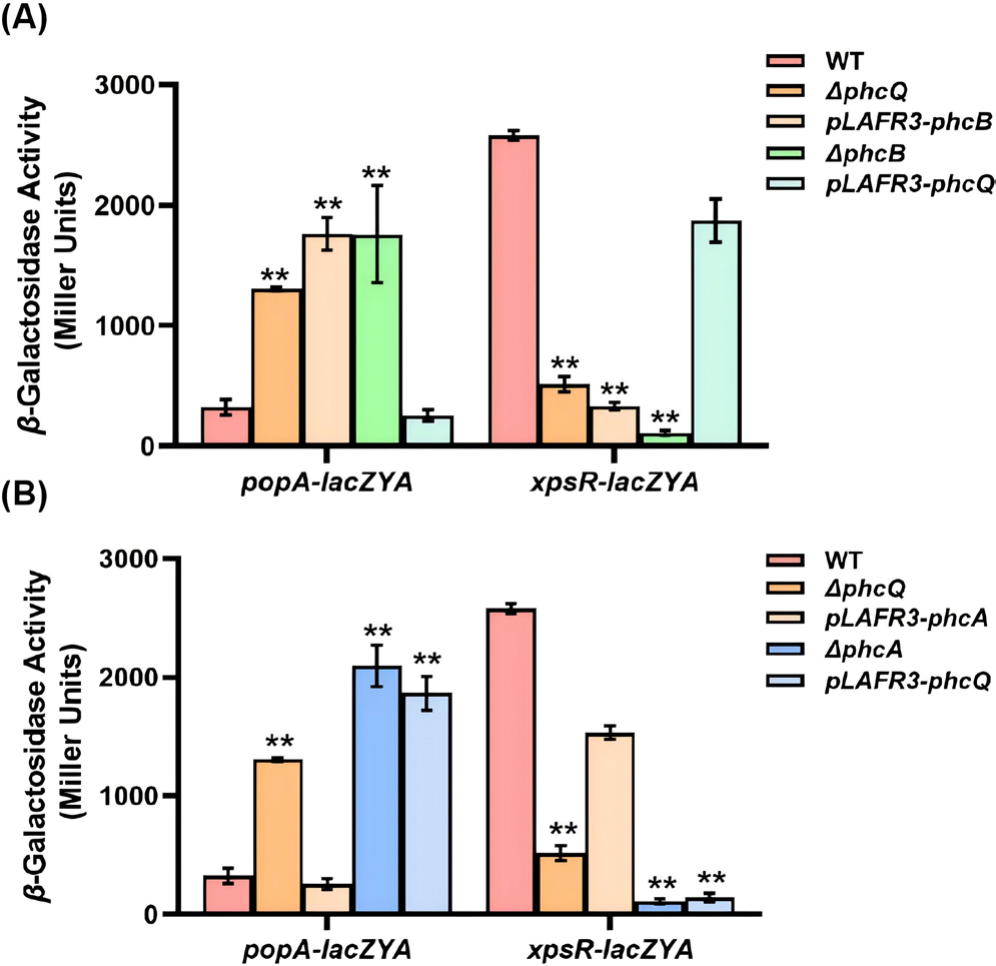
Locations of PhcQ and PhcA within the quorum‐sensing (QS) regulatory pathway using an overexpression system based on the low‐copy number cosmid pLAFR3. (A) Location among PhcQ and PhcB; (B) location among PhcQ and PhcA. Wild type (WT), reporter strains of RK5050 (OE1‐1, *popA‐lacZYA*) or RK5138 (OE1‐1, *xpsR‐lacZYA*); Δ*phcB*, Δ*phcQ* and Δ*phcA* refer to deletion mutants from corresponding reporter strains; pLAFR3‐*phcB*, pLAFR3‐*phcQ* and pLAFR3‐*phcA* refers to overexpression of *phcB*, *phcQ* and *phcA* based on pLAFR3. Plasmids of pLAFR3‐*phcB*, pLAFR3‐*phcQ* and pLAFR3‐*phcA* were introduced into each mutant and expression of *popA‐lacZYA* and *xpsR‐lacZYA* was assessed with enzymatic assay. Each assay was performed with three biological replicates including three replicates per trial. Mean values of all experiments were averaged with SD. Statistical significance was assessed using a post hoc Dunnett test following ANOVA. **p < 0.01.

PhcB, PhcA and PhcQ displayed similar behaviours in regulating expression of PhcA‐controlled QS genes. To ascertain their positions within the QS regulatory pathway, we used an overexpression system based on the low‐copy number cosmid pLAFR3 (Keen et al. 1988). Deleting *phcB* or *phcA* significantly increased expression levels of *popA‐lacZYA* (T3SS), but decreased expression levels of *xpsR‐lacZYA* (EPS) (Clough et al. 1997; Huang et al. 2024). PhcQ was firstly overexpressed in the *phcB* deletion background that substantially compensated for effects caused by *phcB* deletion: it substantially decreased *popA* expression but increased *xpsR* expression (Figure 5A). Conversely, it had no such effects in the *phcA* deletion background (Figure 5B). Overexpression of *phcB* was independent from effects caused by *phcQ* deletion (Figure 5A), indicating that PhcQ is located downstream of PhcB.

PhcA is well known to be located downstream of PhcB, and it was overexpressed in *phcQ* deletion background (Figure 5B). Overexpression of *phcA* compensated for effects caused by *phcQ* deletion: it substantially decreased *popA* expression, but increased *xpsR* expression (Figure 5B), confirming that PhcA is located downstream of PhcQ. All of these results confirmed that PhcQ is located downstream of PhcB, but upstream of PhcA.

### 2.5 PhcQ Is Important to Maintain Protein Stability of PhcA

**FIGURE 6:**
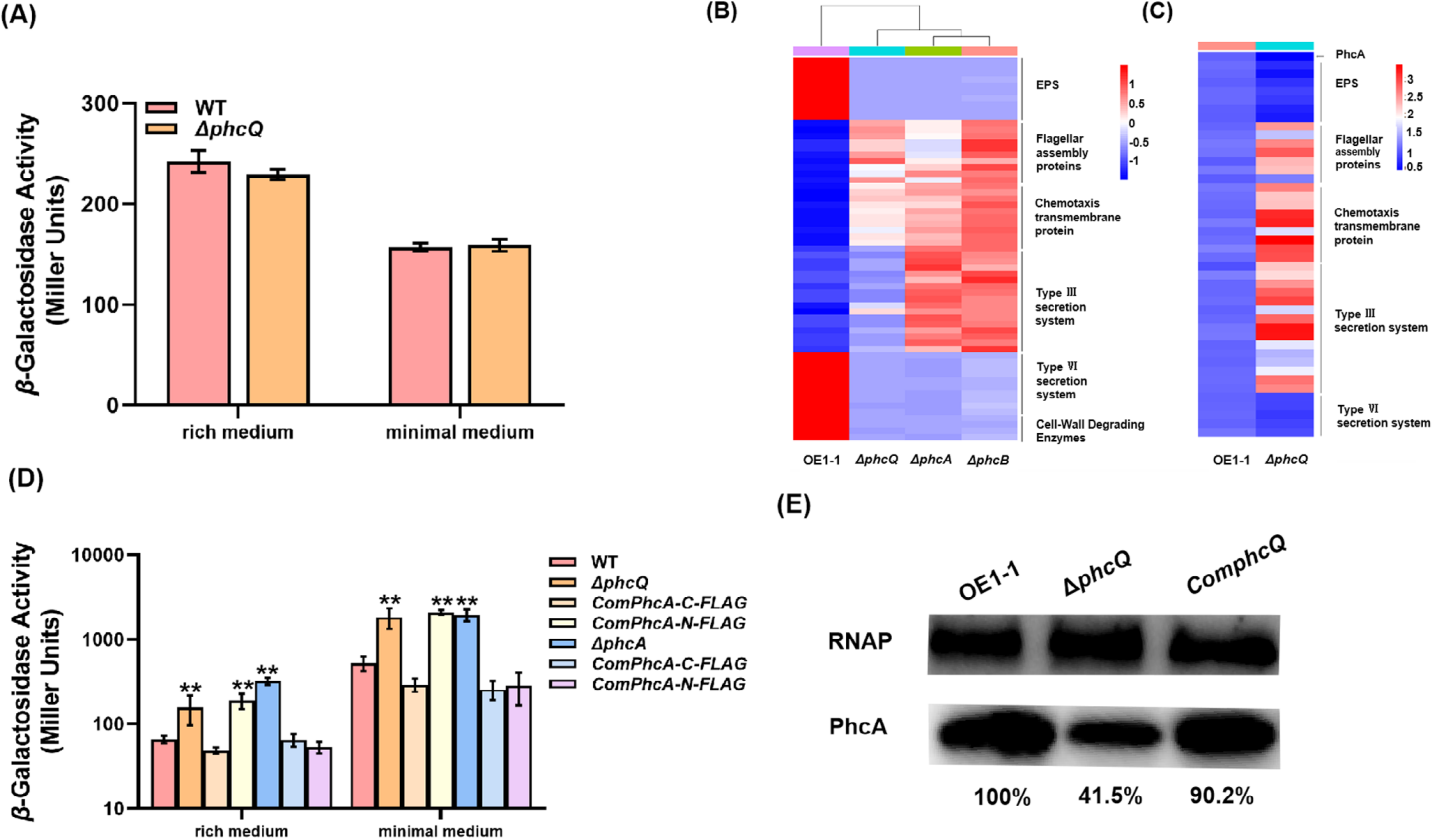
Impact of PhcQ on *phcA* expression and PhcA stability. (A) Effect of PhcQ on *phcA‐lacZYA* expression in rich and minimal medium. (B, C) Heatmap visualisation of differential expression of quorum‐sensing (QS)‐related genes across the transcriptomic and proteomic datasets. (D) Impact of FLAG tag fused at N‐terminus or C‐terminus on PhcA function. (E) Impact of PhcQ on the PhcA stability with western blot. Wild type (WT), RK5043 (*phcA‐lacZYA*); Δ*phcQ*, RQ6322 (*phcA‐lacZYA*, Δ*phcQ*). Strains were grown to 0.1 OD_600_ and subjected to the enzymatic assay. Each assay was performed with three biological replicates including three replicates per trial. Mean values of all experiments were averaged with SD. Statistical significance was assessed using a post hoc Dunnett test following ANOVA. **p < 0.01.

PhcQ regulates expression of QS‐dependent genes mediated through PhcA, whereas deletion of *phcQ* did not affect expression levels of *phcA‐lacZYA* in either rich medium or *hrp*‐inducing minimal medium (Figure 6A). We further assessed protein stability of PhcA with proteomic experiments and western blot. Our previous transcriptomics analysis demonstrated that deletion of either *phcQ* or *phcA* significantly decreased transcription of genes encoding EPS synthesis and the type VI secretion system (T6SS), but significantly increased those for the T3SS apparatus, flagellar assembly and chemotaxis (Figure 6B, Takemura et al. 2021). The tandem mass tag proteomic analysis suggested that *phcQ* deletion significantly increased amounts of proteins involved in the T3SS apparatus, flagellar assembly and chemotaxis, but significantly increased amounts of proteins involved in EPS synthesis and the T6SS (Figure 6C). It was fully consistent with results from the RNA‐seq. Notably, the amount of PhcA protein was significantly reduced with *phcQ* deletion although *phcA* transcription was not altered with *phcQ* deletion (Figure 6A,B). This indicated that some novel protease might begin to degrade PhcA in the absence of PhcQ, and PhcQ might prevent this protease from degrading PhcA.

For the western blot, the FLAG tag was fused to PhcA along with its native promoter and integrated into the chromosome using the Tn*7*‐based chromosomal integration system. Consequently, PhcA‐FLAG could be constitutively expressed in *Ralstonia* cells and the amount of PhcA protein was assessed with western blot. We first evaluated the FLAG‐tag at either the N‐terminus or the C‐terminus to determine its optimal position for PhcA using the complementation assay. The FLAG‐tag at the C‐terminus (com‐phcA‐C‐FLAG) or N‐terminus of PhcA (com‐phcA‐N‐FLAG) fully complemented the phenotype of *phcA* mutants (Figure 6C). Intriguingly, the FLAG‐tag at the C‐terminus of PhcA (com‐phcA‐C‐FLAG) fully complemented the phenotype of *phcQ* mutants to that of the wild‐type strain, but not that at the N‐terminus (com‐phcA‐N‐FLAG) (Figure 6C), indicating that the FLAG‐tag at the PhcA C‐terminus might alter its functional role, possibly protein stability. The FLAG‐tag at the N‐terminus was preferably subjected to western blot to assess the amount of PhcA. Consistent with the aforementioned proteomic results, the amount of PhcA‐FLAG was significantly reduced to approximately 49.5% in *phcQ* mutants compared to the wild type (Figure 6D), confirming that some novel protease begins to degrade PhcA in the absence of PhcQ, and PhcQ helps to maintain the protein stability of PhcA.

### 2.6 The PhcA C‐Terminus Is Important for Mediating Physical Interaction Between PhcQ and PhcA

**FIGURE 7:**
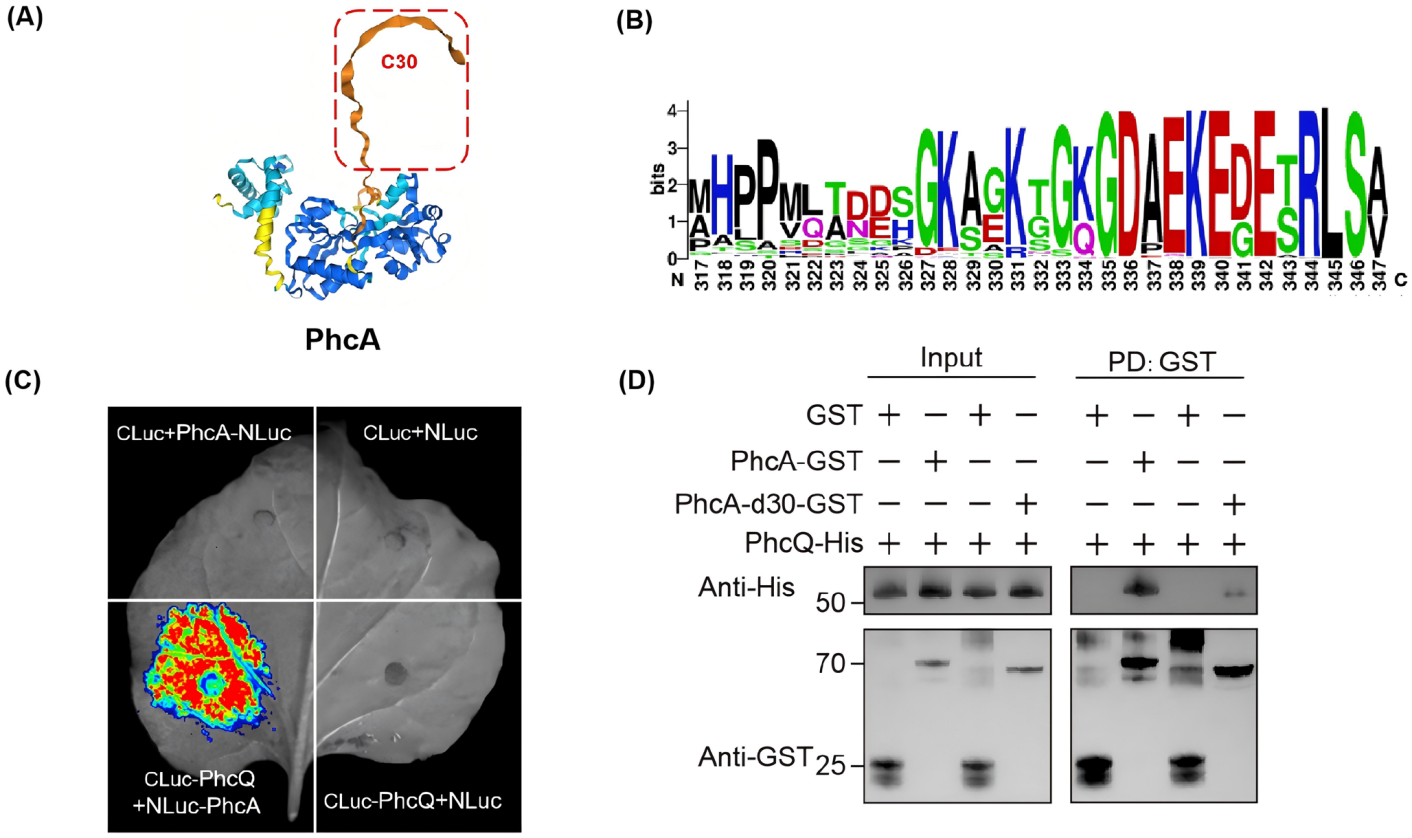
Structural analysis of PhcA and evaluation of its C‐terminal disordered tail mediated physical interaction between PhcQ and PhcA. (A) Predicted structure of PhcA in *Ralstonia pseudosolanacearum* OE1‐1 was generated using AlphaFold2 and visualised with PyMOL. Red dashed‐line circle highlights the C‐terminal disordered tail. (B) Evolution analysis of the C‐terminal regions of PhcA with a bit‐score. (C, D) Physical interaction between PhcQ and PhcA using the bimolecular fluorescence complementation assay in *Nicotiana benthamiana* leaves (C) and using the glutathione S‐transferase (GST) pull‐down assay (D). PhcA‐d30 refers to truncated PhcA at its C‐terminal disordered tail with 30 amino acids (AA). Each assay was performed with three biological replicates and representative pictures were presented.

**FIGURE 8:**
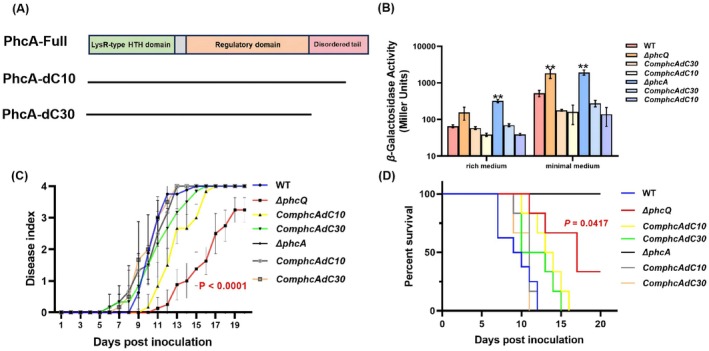
Functional evaluation of C‐terminus disordered tail in PhcA. (A) Diagram of truncated PhcA at C‐terminus with 10 amino acids (PhcA‐dC10) and 30 amino acids (PhcA‐dC30). (B) Functional evaluation of truncated PhcA on *popA‐lacZYA* expression and (C, D) virulence test in tobacco plants with inoculation method of the soil‐soaking (C, disease index; D, plant survival curves). Wild type (WT), RK5050 (OE1‐1, *popA‐lacZYA*); Δ*phcQ*, RQ6289 (*popA‐lacZYA*, Δ*phcQ*); Δ*phcA*, RK6820 (*popA‐lacZYA*, Δ*phcA*); *ComphcAdC30*, RQC1889 (complementation of truncated PhcA‐d30 into RQ6820); *ComphcAdC10*, RQC2029 (complementation of truncated PhcA‐d10 into RQ6820). For the enzyme assay, each assay was performed with three biological replicates, each including three technical replicates. For virulence test, each assay was performed with three biological replicates and each biological replicate includes 12 plants. Mean values of all experiments were averaged with SD. Statistical significance between mutants and parent strains was assessed using a post hoc Dunnett test following ANOVA. **p < 0.01.

Through structural analysis, the C‐terminus of PhcA, comprising approximately 30 amino acids, lacks a defined three‐dimensional structure. It forms a disordered tail that may make it susceptible to degradation (Figure 7A). An alignment of the C‐terminal 30 amino acids of PhcA was generated from 76 RSSC strains, and sequence similarity was assessed using bit scores. The last dozen amino acids are significantly more conserved than those in the preceding segment (Figure 7B). PhcA was therefore truncated at the C‐terminal 10 (PhcA‐dC10) or 30 amino acids (PhcA‐dC30) and complemented into *phcA* and *phcQ* mutants to evaluate their impact on PhcA function (Figure 8A). Both truncated PhcA versions substantially restored T3SS expression (Figure 8B) and virulence in *phcA* mutants (Figure 8C,D), indicating that this C‐terminal disordered tail is not required for PhcA function. Intriguingly, under *phcQ* deletion, complementation of truncated PhcA (PhcA‐dC30 and PhcA‐dC10) substantially decreased T3SS expression, which was increased by *phcQ* deletion (Figure 8C), and also substantially restored virulence impaired by *phcQ* deletion (Figure 8C,D), indicating that the truncated PhcA might enhance protein stability in the absence of PhcQ.

We investigated whether PhcQ protected PhcA from degradation by direct binding. First, a bimolecular fluorescence complementation (BiFC) assay was conducted to test their direct physical interaction in *Nicotiana benthamiana* leaves. Following *Agrobacterium*‐mediated transient co‐expression of PhcA and PhcQ, clear fluorescence was detected, confirming a direct physical interaction between the two proteins in the leaves (Figure 7C). The in vitro direct binding was further investigated using a glutathione S‐transferase (GST) pull‐down assay. The results confirmed that PhcA‐GST, but not GST alone, directly binds to PhcQ‐His (Figure 7D). Intruingly, direct binding was also observed between truncated PhcA (PhcA‐dc30‐GST) and PhcQ‐His, though the amount of PhcQ‐His pulled down by the truncated PhcA was significantly reduced (Figure 7D). All of these results indicated that PhcQ binds directly to PhcA and thus protected it from degradation. The disordered tail in the PhcA C‐terminus plays an important role in their direct physical interaction.

### 2.7 PhcA Stability Is Not Governed by Typical Proteases or Omics‐Selected Candidates

**FIGURE 9:**
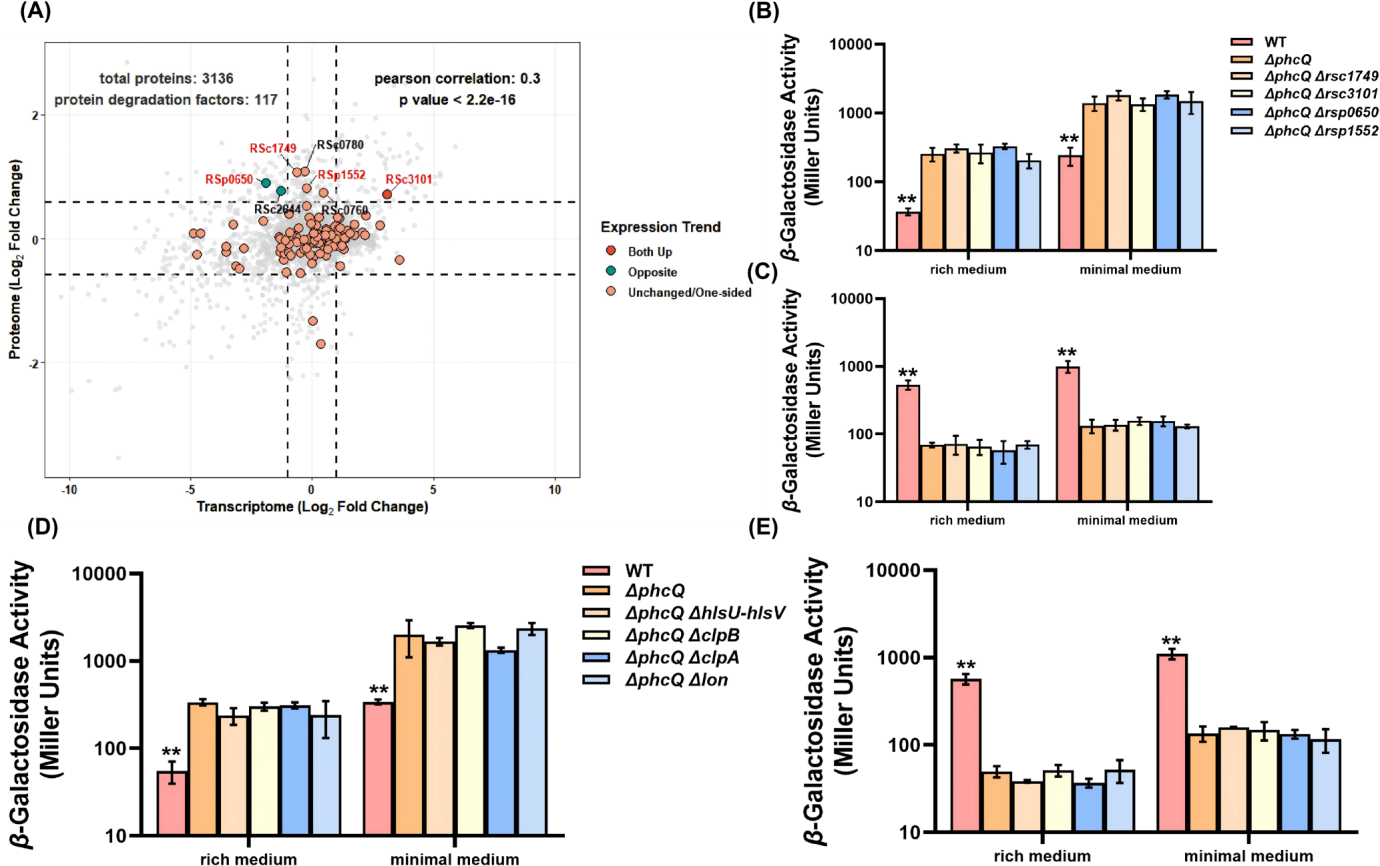
Functional evaluation of proteases or omics‐selected candidate proteases on PhcA. (A) Correlation analysis by comparing log_2_ fold changes in gene expression and protein abundance (integrated transcriptomic and proteomic data, *phcQ* mutant versus wild type). Enriched 3316 proteins across both datasets are presented as grey dots. 117 putative proteases or protease‐related factors were selected based on functional annotation and presented in colour dots (red, green and orange). Seven proteins (significantly up‐regulated based on proteomic data) are listed with protein names. RSc3101 is presented as a red dot (both up) because it was significantly up‐regulated in both datasets. RSp0650 and RSc2644 are presented in green dots (opposite) because they were significantly up‐regulated from proteomic data but down‐regulated from transcriptomic data. (B–E) Functional evaluation of four candidates (RSc1749, RSc3101, RSp0650 and RSp1552) and typical proteases (Lon, HslVU, ClpA and ClpB) on PhcA controlling expression of *popA* (B, D) and *xpsR* (C, E) in rich and minimal medium. Wild type (WT), RK5050 (OE1‐1, *popA‐lacZYA*); Δ*phcQ*, RQ6289 (*popA‐lacZYA*, Δ*phcQ*); Δ*rsc1749*, Δ*rsc*3101, Δ*rs*p0650, Δ*rs*p1552, Δ*rsc1713* (*lon*), Δ*rsc0042‐0043* (*hslVU*), Δ*rsc2464* (*clpA*) and Δ*rsc1335* (*clpB*) refer to deletion mutants from RQ6289. The enzyme assay was performed with three biological replicates, including three replicates per trail. Mean values of all experiments were averaged with SD. Values of Δ*phcQ* strains served as the controls and *p*‐values calculated in comparison to these controls using a post hoc Dunnett test following ANOVA. **p < 0.01.

To identify novel proteases that degrade PhcA protein, we conducted a correlation analysis comparing the log_2_ fold changes in gene expression and protein abundance using integrated transcriptomic and proteomic data (*phcQ* mutant versus wild‐type). Of these, 3136 proteins were enriched across both datasets and 117 proteins were selected as putative proteases or protease‐related factors based on their functional annotation (dots in three colours, Figure 9A). From this pool, seven proteins were significantly up‐regulated based on proteomic data (listed with protein names, Figure 9A). Note that RSp0650 and RSc2644 were significantly down‐regulated from transcriptomic data (green dots), and RSc3101 was significantly up‐regulated in both datasets (red dot). In this study, four candidates of RSc1749, RSc3101, RSp0650 and RSp1552 (with red labels) were selected to evaluate whether they were involved in PhcA function.

We deleted these four genes from reporter strains of RQ6289 (*popA‐lacZYA*, Δ*phcQ*) and RQ6326 (*xpsR‐lacZAY*, Δ*phcQ*) to determine whether they alter PhcA function in the absence of PhcQ. Deletion of *phcQ* substantially increased *popA* expression but decreased *xpsR* expression in both rich and minimal media (Figure 9B,C). Deletion of either gene exhibited identical expression levels of *popA* and *xpsR* to that in the *phcQ* mutant (Figure 9B,C).

In bacteria, several ATP‐dependent proteases, that is, Lon, HslVU and the Clp family proteins, execute intracellular proteolysis (Zhou et al. 2018; Baytshtok et al. 2021; Hoskins et al. 1998). We investigated whether these proteases are involved in PhcA function. Deletion of *rsc1713* (*lon*), *rsc0042‐0043 (hslVU), rsc2464* (*clpA*) or *rsc1335* (*clpB*) did not alter expression levels of *popA* and *xpsR*, even in the absence of PhcQ (Figure 7A,B), indicating that the stability of PhcA was not governed by these typical proteases or some omics‐selected candidates.

## 3 Discussion

In the present study, we provided multiple lines of evidence to demonstrate that PhcQ acts as a key intermediary stabiliser of PhcA. It emphasises the importance of post‐translational regulation in the Phc QS system mediated through the central regulator PhcA and offers novel insights into intricate virulence control mechanisms in RSSC. Previous models of the Phc QS system established PhcB as the signal generator and PhcA as the central transcriptional regulator. However, the functional gap between PhcB and PhcA activation was not fully resolved (Genin and Denny 2012; Kai 2023). PhcA directly binds to promoters of *xpsR* and *prhIR* and regulates their expression positively or negatively, respectively (Huang et al. 1998; Yoshimochi, Hikichi, et al. 2009; Yoshimochi, Zhang, et al. 2009). The phenotypic consequences of *phcQ* deletion, namely enhanced T3SS expression, reduced EPS production, accelerated early growth, and attenuated virulence, perfectly mirror those of a *phcA* deletion. Further experiments provided particularly compelling evidence: overexpression of *phcA* could phenotypically compensate for the loss of *phcQ*, but overexpression of *phcQ* could not rescue that of a *phcA* mutant. These genetic epistasis experiments solidify PhcQ's position within the Phc QS hierarchy: it acts downstream of PhcB but upstream of PhcA. PhcQ regulates the expression of these QS‐dependent genes mediated through PhcA.

The most striking finding of this study is the dramatic reduction of PhcA protein levels (to approximately 40% of the wild type) in the *phcQ* mutant, as consistently confirmed by both proteomic analysis and western blotting, despite the unchanged transcriptional level of *phcA*, which unambiguously positions PhcQ as a crucial stabiliser of PhcA protein. It indicates that the primary, if not sole, role of PhcQ is to ensure sufficient levels of PhcA protein to properly execute the Phc QS programme. These refines the definition of upstream regulation in this context. Unlike typical cascades where one regulator affects the transcription of the next one, PhcQ is a critical post‐translational regulator maintaining the stability of PhcA, rather than influencing the transcription. These results revealed PhcQ as bridging the functional gap between PhcB and PhcA activation within the Phc QS regulatory cascade.

Note that *phcA* mRNA levels remained unchanged in *phcQ* mutants, while protein levels of PhcA were dramatically decreased. This uncoupling of transcription and translation strongly implies that PhcA is subject to constitutive degradation and that PhcQ acts to counteract this process. Note that the low‐cell‐density‐mode mimicked the phenotype of *phcA* mutants during infection through roots (Khokhani et al. 2017), whereas expression levels of *phcA* remained relatively stable during low and high cell densities (Figure 6). PhcA becomes active at high cell densities (Clough et al. 1997), and binds to the promoter of *prhIR* and shuts down its transcriptional expression when cell density reaches a specific threshold (Yoshimochi, Hikichi, et al. 2009). We propose a possibility that the distinct regulatory behaviour of PhcA at different cell densities may result from insufficient protein levels at the low cell density. Regulating the master regulator PhcA through maintaining protein stability, rather than transcription, offers distinct evolutionary advantages. If PhcA protein levels were controlled solely by transcription, the bacterium would face a time lag during *de novo* synthesis or mRNA decay. In contrast, a strategy of constitutive degradation and conditional stabilisation switching allows for a much faster response to environmental changes than transcriptional activation alone (Battesti and Gottesman 2013; Jenal and Hengge‐Aronis 2003).

To identify which protease mediates the degradation of PhcA, we conducted a correlation analysis by integrating transcriptomic and proteomic datasets and selected several putative proteases with significant up‐regulation from both datasets (Figure 9A). Together with four well‐studied typical ATP‐dependent proteases, Lon, HslVU, ClpA and ClpB (Hoskins et al. 1998; Zhou et al. 2018; Baytshtok et al. 2021), which were not enriched from the correlation analysis, eight putative protease genes were deleted to test their involvement in PhcA function, especially in the *phcQ* deletion background. No results ruled out these major proteolytic systems as mediators of PhcA instability in the absence of PhcQ. It points towards the involvement of a more specialised or novel protease system that specifically targets PhcA. It was confirmed that PhcQ binds directly to PhcA both in vitro and *in planta* using the BiFC and GST‐pull down assays. Structural analysis of PhcA provides a crucial clue: the presence of a disordered C‐terminal tail, a feature that often serves as a ‘degron’ signal‐specific motif recognised by the proteolytic machinery to ensure rapid turnover (Zhang et al. 2025). This disordered C‐terminal tail is dispensable for PhcA function yet is critical for protein stability because truncation of PhcA at this tail (ΔC10, ΔC30) restored PhcA function in the absence of PhcQ. Moreover, it impaired the binding affinity of PhcQ and PhcA. It is plausible that PhcQ may function by binding to or masking this degradation‐prone region, thereby preventing its recognition by a putative, yet‐unidentified protease. Future work will focus on identifying this protease through pull‐down assays or screening suppressor mutants to elucidate the precise mechanism by which PhcQ antagonises its activity.

The *phcQ* mutant mimics the *phcA* mutant phenotype—high T3SS expression (invasion mode) and low EPS (colonisation mode). This explains why the *phcQ* mutant grows rapidly in early stages and outcompetes the wild type initially (high motility/invasion) but fails to cause rapid wilting (low biomass/clogging ability). Although the *phcQ* mutant completely lost virulence on tomato plants, it retained weak delayed virulence on tobacco plants (Takemura et al. 2021). This suggests that different hosts require different thresholds of PhcA activity. The initial boost in T3SS and motility might grant the *phcQ* mutant a slight advantage during early invasion in some hosts (like tobacco plants), facilitating limited proliferation. Tobacco plants may be more susceptible to the low levels of PhcA remaining in the *phcQ* mutant, or the immune response in tomato plants might require a more robust EPS shield that the mutant lacks. This is supported by the competitive index data showing a slight initial *in planta* advantage. However, the critical inability to produce EPS, a key virulence factor for vascular clogging and wilting in later stages, ultimately cripples its pathogenicity, especially in more resistant hosts like tomato plants (Milling et al. 2011). This host‐dependent phenotypic outcome underscores the complex interplay between pathogen virulence strategies and host susceptibility.

In summary, our findings define a novel regulatory layer in the QS circuitry where PhcQ stabilises the master regulator PhcA. By preventing the premature degradation of PhcA mediated by its C‐terminal disordered region, PhcQ ensures the precise timing of virulence expression. This study not only clarifies the molecular function of PhcQ but also highlights protein stability as a pivotal control point in bacterial pathogenesis, offering potential new targets for antivirulence strategies.

## 4 Experimental Procedures

### 4.1 Bacterial Strains and Growth Condition

Bacterial strains used in this study are listed in Table S1. *Escherichia coli* strains DH12S and S17‐1 were grown in Luria Bertani (LB) medium at 37°C and used for plasmid construction and conjugational transfer, respectively. *R. pseudosolanacearum* strains were grown at 28°C in a broth medium (rich medium) or a minimal medium (Hoagland medium with 2% of sucrose, also used as a *hrp*‐inducing medium) (Yoshimochi, Zhang, et al. 2009). These strains were derivatives of OE1‐1, a phylotype I, race 1 and biovar 4 pathogenic strain on tomato and tobacco plants (Kanda et al. 2003).

### 4.2 Generation of Mutants With Gene Deletion, Complementation and Overexpression

Mutants were generated with in‐frame deletion of target genes using pK18mobsacB based homologous recombination as previously described, which is marker‐free and appropriate for multiple deletion of a subset of genes (Zhang et al. 2015; Lei et al. 2020). Genetic complementation was performed with a Tn*7*‐based site‐specific chromosomal integration system as described previously, which ensures constitutive expression of target genes with their native promoters (Zhang et al. 2021). Overexpression was carried out using the low copy number cosmid pLAFR3 (Keen et al. 1988). Briefly, target genes were PCR amplified with their putative native promoters and cloned into pLAFR3. After sequence verification, plasmids of pLAFR3‐*phcB*, pLAFR3‐*phcQ* and pLAFR3‐*phcA* were introduced into each mutant by electroporation, and positive transformants were selected on agar plates with appropriate antibiotics.

### 4.3 Enzymatic Assay for *β*‐Galactosidase

The expression of genes fused with promoterless *lacZYA* was assessed by enzymatic assay for *β*‐galactosidase as previously described, enzyme activities of which in medium were expressed in Miller Units (Miller 1992; Zhang et al. 2013). Each assay was performed with three biological replicates and three replicates per trial. Mean values of all experiments were averaged with SD. Statistical significance was assessed using a post hoc Dunnett test following ANOVA.

### 4.4 Bacterial Growth Assay and Competitive Fitness Assay

Bacterial growth was assessed both in vitro (rich medium and minimal medium) and *in planta* (tobacco leaves) as previously described (Zhang et al. 2017). The in vitro growth was evaluated by monitoring an optical density at 600 nm (OD_600_) using an automatic microbial growth curve analyser (MGC‐500; SCIENTZ). Briefly, overnight cultivation of the wild‐type strain (OE1‐1), mutants of Δ*phcQ*, Δ*phcA* or Δ*phcB*, and complemented strains was resuspended in fresh medium and adjusted to an OD_600_ of 0.01. Cell suspension was transferred into a microplate containing 200 μL, and growth curves were recorded automatically with shaking at 28°C. Each assay was performed with three biological replicates, including four replicates per trial. The maximum growth rate and biomass, lag‐phase time and area under the curve were determined using the *gcFitModel* function to fitgrowth data with the *grofit* R package v. 1.1.1 (Jiang et al. 2024). For growth in leaves, cell suspension at 10^4^ cfu mL^−1^ was infiltrated into tobacco leaves, and cells densities were quantified by dilution plating, which was represented in log_10_ CFU cm^−2^. For competitive fitness assay, the wild‐type strain and *phcQ* mutants were marked with genes of Gm^r^ (gentamycin resistance) and Km^r^ (kanamycin resistance), respectively, as previously described (Huang et al. 2024). The Km^r^ and Gm^r^ strains at 10^4^ cfu mL^−1^ were mixed at 1:1 and infiltrated into tobacco leaves. Bacteria recovered from leaves were diluted 10‐fold and simultaneously spread onto agar plates with Km or Gm. Cell numbers of Km^r^‐ and Gm^r^‐resistant cells were counted and cell densities in leaves are presented in log_10_ CFU cm^−2^. The ratio between the wild‐type strain and *phcQ* mutants were also calculated. Each assay in tobacco leaves was performed with four biological replicates, including six plants per trial. Mean values of all experiments were averaged with SD. Statistical significance was assessed using a post hoc Dunnett test following ANOVA.

Bacteria morphology was also observed on agar plates. About 100 μL of cell suspension at 10^3^ cfu mL^−1^ was spread onto broth agar plates. Morphology of single colonies was photographed after 48 h. All observations were repeated three times and representative morphological results were presented.

### 4.5 Virulence Assay

Virulence assay was performed on susceptible tobacco plants ( *Nicotiana tabacum* ‘Bright Yellow’). Plants were grown at 25°C for about 4–5 weeks and subjected to inoculation with the soil‐soaking method mimicking natural invasion through roots and leaf infiltration, enabling direct invasion into intercellular spaces of leaves.

Each assay was performed with four biological replicates, including 12 plants per trial. Wilt symptoms of plants were inspected daily as 1–4 disease indices (Roberts et al. 1988). The virulence test was presented with survival curves, by which test plants were inspected with two levels of no symptoms (disease index below 3) and complete wilting (disease index equal to or higher than 3) as previously described (Poueymiro et al. 2009). The statistical significance was assessed using a post hoc Dunnett test following ANOVA.

### 4.6 Transcriptome Sequencing

A previously published RNA‐seq dataset generated by Takemura et al. (2021) was used in this study. The processed expression matrix (Excel format), which includes normalised gene expression values for *R. pseudosolanacearum* strains OE1‐1, Δ*phcB*, Δ*phcA* and Δ*phcQ* grown in quarter‐strength M63 medium, was obtained directly from the Table S2 of the original publication. Heatmaps were generated in R using the *ComplexHeatmap* package (v. 2.14.0).

### 4.7 Proteomics Analysis

Cells of the wild‐type strain and mutant were grown in rich medium and harvested at logarithmic phase. Cell pellet was washed twice with ice‐cold phosphate‐buffered saline (PBS), then resuspended in ice‐cold lysis buffer (2 M thiourea, 7 M urea, 4% CHAPS, 30 mM Tris pH 8.5) containing 100 μL of protease inhibitor cocktail (Sigma‐Aldrich) with stirring at 4°C for 30 min. The lysate was centrifuged at 15,000 *g* at 4°C for 30 min. Supernatants were saved as the whole cell extract and subjected for proteomic analysis with an Orbitrap Astral high resolution mass spectrometer (Majorbio Co. Ltd.). A subset of genes, identified as significantly differentially expressed from the RNA‐seq analysis, were selected for protein‐level analysis based on the proteomic results. Each assay was performed with three biological replicates, representing three independent cultivation processes. The proteomic results from these replicates were averaged and subjected for robust protein‐level analysis. The data were analysed through the free online platform of the Majorbio Cloud Platform (cloud.majorbio.com). Heatmaps were generated using the *ComplexHeatmap* package (v. 2.14.0) with default settings and customised colour scales.

### 4.8 Western Blot Analysis for Protein Expression and Stability

The FLAG tag (DYKDDDDK) was fused to either the N‐terminus or C‐terminus of the PhcA protein, and the stability of PhcA was evaluated with western blot analysis. Briefly, a DNA fragment containing the *phcA* native promoter, coding sequence, and 3 × FLAG was cloned into pUC18‐mini‐Tn*7*T‐Gm. The *phcA‐FLAG* was thus integrated into the chromosome via this Tn*7*‐based site‐specific chromosomal integration system that ensures constitutive expression of *phcA‐FLAG* in the wild‐type strain and *phcQ* mutants. Cells were grown in rich medium and harvested at logarithmic phase. SDS‐PAGE analysis of cell lysates and immunoblotting were performed using standard techniques; the signal was visualised and quantified using the FUSION Pulse TS imaging system (Vilber) with ECL substrate and the ImageJ Analysis Software. Primary antibodies, including anti‐FLAG (Sigma‐Aldrich) and RNA polymerase β‐subunit (used as a loading control), were diluted in Tris‐buffered saline with Tween 20 (TBST) as appropriate. Horseradish peroxidase (HRP)‐conjugated goat anti‐rabbit IgG (Sigma‐Aldrich) was used as the secondary antibody and diluted according to the instructions. Each assay was performed with three biological replicates, and representative results are presented.

### 4.9 Correlation Analysis Using Integrated Transcriptomic and Proteomic Data

To conduct correlation between transcriptomic and proteomic datasets, two datasets were aligned using matching locus tags, and the log_2_ fold change (FC) values of co‐quantified features were compared. Genes related to protein degradation were identified based on annotation keywords, including protease, peptidase, degradation, clp, lon and ftsH. Genes were categorised into different expression trends using thresholds of |log_2_FC| ≥ 1.0 for mRNA and |log _2_FC| ≥ 0.58 for proteins (1.5‐FC in protein abundance). Genes showing coordinated up‐regulation or down‐regulation were classified as ‘Both Up’ or ‘Both Down’, respectively. In contrast, genes exhibiting discordant changes at the transcriptomic and proteomic levels were categorised as ‘Opposite’. The remaining genes were classified as ‘Unchanged/One‐sided’. Pearson correlation analysis was performed to evaluate the concordance between transcriptomic and proteomic changes, and visualisation was carried out in R (v. 4.2.1) using the ggplot2 and ggrepel packages.

### 4.10 Luciferase Complementation Imaging Assay

The luciferase complementation imaging assay was conducted following previously described (Luo et al. 2025). Coding sequences of *phcA and phcQ* were cloned into NLuc‐BS vector and CLuc‐BS vector, respectively, and subsequently transformed into *Agrobacterium tumefaciens* GV3101. GV3101 strains harbouring two vectors were mixed in equal proportions and infiltrated into leaves of 4‐ to 5‐week‐old *Nicotiana benthamiana* plants. At 36 to 48 h post‐infiltration (hpi), the infiltrated areas were treated with 0.1 mM luciferin and kept in the dark for 5 min. Images were captured using the plant living imaging system (VILBER, NEWTON7.0 Bio plus). Each assay was conducted with three biological replicates, including four leaves per trial. Representative images are presented.

### 4.11 GST Pull‐Down Assay

The GST pull‐down assay was conducted following the previously description (Luo et al. 2025). In brief, coding sequences of *phcA* and *phcQ* were cloned into pGEX‐4T‐1‐BS and pET‐32a(+)‐BS, respectively, and expressed in *E. coli* BL21 under IPTG induction at 16°C. The concentrations of purified fusion proteins (PhcA‐GST, PhcAd30‐GST and PhcQ‐his) were measured and equal amounts of fusion proteins were mixed for the GST pull‐down assay. Proteins were separated with SDS‐PAGE and detected with the immunoblotting. Each assay was performed with three biological replicates and representative pictures were presented.

## Author Contributions

**Huixin Yang:** methodology, data curation. **Lili Luo:** investigation, data curation, methodology. **Tao Guo:** investigation, methodology, data curation, supervision. **Kouhei Ohnishi:** conceptualization, writing – review and editing, data curation. **Yong Zhang:** conceptualization, writing – review and editing, methodology, data curation, supervision, funding acquisition. **Min Chen:** writing – original draft, data curation, investigation, methodology. **Yasufumi Hikichi:** conceptualization, writing – review and editing, supervision. **Liujie Hu:** funding acquisition, data curation, validation, methodology. **Tao Liang:** investigation, data curation, validation, funding acquisition, supervision. **Chengtao Li:** conceptualization, investigation, data curation, writing – review and editing, supervision. **Masayuki Tsuzuki:** conceptualization, writing – review and editing, supervision. **Peng Li:** methodology, investigation, writing – review and editing.

## Funding

This work was supported by the National Natural Science Foundation of China (32570216 and 32170180 to Y. Z.; 32260652 to P. L.), the Chongqing Bureau of Science and Technology (CSTB2022TIAD‐CUX0020) to T. L. and the Chongqing Academy of Agricultural Sciences (KYLX20240500097) to L. H.

## Conflicts of Interest

The authors declare no conflicts of interest.

## Supporting information


**Table S1:** Bacterial strains used in this study.

**Table S2:** Primers used in this study.

## Data Availability

The data that support the findings of this study are available on request from the corresponding author. The data are not publicly available due to privacy or ethical restrictions.
