# Supplementary material for: Ralstonia pseudosolanacearum PhcQ Controls Quorum Sensing‐Dependent Phenotypes by Binding PhcA and Maintaining Its Protein Stability
Source: Mol Plant Pathol. 2026 Jul 1;27(7):e70308. doi: 10.1111/mpp.70308 (PMC13320629; doi:10.1111/mpp.70308)
Supplement: Supplementary file 1 — Table S1: Bacterial strains used in this study. [file MPP-27-e70308-s001.docx]

**Table S1. Bacterial strains used in this study**

| Strain | Relative characteristics | References |
| --- | --- | --- |
| OE1-1 | Wild-type, race 1, biovar 3 | Kanda *et al*., 2003 |
| RK5043 | OE1-1, *phcA-lacZYA* | Yoshimochi *et al*., 2009 |
| RK5046 | OE1-1, *hrpB-lacZYA* | Yoshimochi *et al*., 2009 |
| RK5050 | OE1-1, *popA-lacZYA* | Yoshimochi *et al*., 2009 |
| RK5120 | OE1-1, *hrpG-lacZYA* | Yoshimochi *et al*., 2009 |
| RK5124 | OE1-1, *prhJ-lacZYA* | Yoshimochi *et al*., 2009 |
| RK5130 | OE1-1, *prhIR-lacZYA* | Yoshimochi *et al*., 2009 |
| RK5134 | OE1-1, *prhA-lacZYA* | Yoshimochi *et al*., 2009 |
| RK5138 | OE1-1, *xpsR-lacZYA* | Yoshimochi *et al*., 2009 |
| RK5212 | OE1-1, *prhG-lacZYA* | Zhang *et al*., 2013 |
| RQ6820 | *popA-lacZYA, ΔphcA* | Huang et al., 2024 |
| RQ6822 | *popA-lacZYA, ΔphcB* | Huang et al., 2024 |
| RQ6289 | *popA-lacZYA, ΔphcQ* | This study |
| RQ6319 | *hrpB-lacZYA*, *ΔphcQ* | This study |
| RQ6361 | *hrpG-lacZYA*, *ΔphcQ* | This study |
| RQ6328 | *prhG-lacZYA*, *ΔphcQ* | This study |
| RQ6373 | *prhJ-lacZYA*, *ΔphcQ* | This study |
| RQ6322 | *phcA-lacZYA*, *ΔphcQ* | This study |
| RQ6364 | *prhIR-lacZYA*, *ΔphcQ* | This study |
| RQ6367 | *prhA-lacZYA*, *ΔphcQ* | This study |
| RQ6325 | *xpsR-lacZYA*, *ΔphcQ* | This study |
| RQ6346 | *hrpB-lacZYA*, *ΔphcA* | This study |
| RQ6340 | *hrpG-lacZYA*, *ΔphcA* | This study |
| RQ6358 | *prhG-lacZYA*, *ΔphcA* | This study |
| RQ6331 | *prhJ-lacZYA*, *ΔphcA* | This study |
| RQ6343 | *prhIR-lacZYA*, *ΔphcA* | This study |
| RQ6370 | *prhA-lacZYA*, *ΔphcA* | This study |
| RQ6671 | *xpsR-lacZYA*, *ΔphcA* | This study |
| RQ6334 | *xpsR-lacZYA*, *ΔphcB* | This study |
| RQ6668 | *RQ6286*, *ΔclpB* | This study |
| RQ6782 | *RQ6325*, *ΔclpB* | This study |
| RQ6734 | *RQ6286*, *ΔclpA* | This study |
| RQ6785 | *RQ6325*, *ΔclpA* | This study |
| RQ6749 | *RQ6286*, *Δlon* | This study |
| RQ6788 | *RQ6325*, *Δlon* | This study |
| RQ6634 | *RQ6286*, *ΔhlsU-hlsV* | This study |
| RQ6791 | *RQ6325*, *ΔhlsU-hlsV* | This study |
| RQ6704 | *RQ6286*, *Δrsc3101* | This study |
| RQ6674 | *RQ6325*, *Δrsc3101* | This study |
| RQ6707 | *RQ6286*, *Δrsc1749* | This study |
| RQ6677 | *RQ6325*, *Δrsc1749* | This study |
| RQ6722 | *RQ6286*, *Δrsp0650* | This study |
| RQ6680 | *RQ6325*, *Δrsp0650* | This study |
| RQ6701 | *RQ6286*, *Δrsp1552* | This study |
| RQ6719 | *RQ6325*, *Δrsp1552* | This study |
| RQC2156 | *RQ6334, pLAFR3-phcQ* | This study |
| RQC2057 | RK5050, pUC18-mini-Tn7T-Km (Km^r^) | This study |
| RQC2112 | RQ6289, pUC18-mini-Tn7T-Gm (Gm^r^) | This study |
| RQC1218 | RQ6289, *ComphcQ* | This study |
| RQC1849 | RQ6820, *ComphcA* | This study |
| RQC1939 | *RK5050, phcA-N-FLAG* | This study |
| RQC2026 | *RQ6289, phcA-N-FLAG* | This study |
| RQC1924 | *RQ6820, phcA-N-FLAG* | This study |
| RQC1774 | *RK5050, phcA-C-FLAG* | This study |
| RQC1827 | *RQ6289, phcA-C-FLAG* | This study |
| RQC1835 | *RQ6820, phcA-C-FLAG* | This study |
| RQC1889 | *RQ6289, phcAdC30* | This study |
| RQC2029 | *RQ6289, phcAdC10* | This study |
| RQC1886 | *RQ6820, phcAdC30* | This study |
| RQC2037 | *RQ6820, phcAdC10* | This study |

**References**

Huang J, Wang R, Zhang Q, Wang C, Liang T, Hikichi Y, Ohnishi K, Jiang G, Guo T, Zhang Y. (2024) Positive regulation of the PhcB neighbouring regulator PrhX on expression of the type III secretion system and pathogenesis in *Ralstonia solanacearum*. Mol Plant Pathol,Jan;25(1):e13398.

Kanda A, Ohnishi S, Tomiyama H, Hasegawa H., Yasukohchi ., Kiba A. et al. (2003) Type III secretion machinery-deficient mutants of *Ralstonia solanacearum* lose their ability to colonize resulting in lossof pathogenicity. Journal of General Plant Pathology, 69, 250-257.

Yoshimochi T, Zhang Y, Kiba A, Hikichi Y. and Ohnishi K. (2009) Expression of *hrpG* and activation of response regulator HrpG are controlled by distinct signal cascades in *Ralstonia solanacearum*. Journal of General Plant Pathology, 75:196-204.

Zhang Y, Chen L, Takehi Y, Kiba A, Hikichi Y and Ohnishi K. (2013) Functional analysis of *Ralstonia solanacearum* PrhG regulating the *hrp* regulon in host plants. Microbiology, 159, 1695-1704.
