# Supplementary material for: Ralstonia pseudosolanacearum PhcQ Controls Quorum Sensing‐Dependent Phenotypes by Binding PhcA and Maintaining Its Protein Stability
Source: Mol Plant Pathol. 2026 Jul 1;27(7):e70308. doi: 10.1111/mpp.70308 (PMC13320629; doi:10.1111/mpp.70308)
Supplement: Supplementary file 2 — Table S2: Primers used in this study. [file MPP-27-e70308-s002.docx]

**Table S2. Primers used in this study**

| primer | sequence | Reference |
| --- | --- | --- |
| phcQA1B | ATGGATCCACACCTATCCGTTCACCGCT | This study |
| phcQB1C | CGGCGCCGCGGGGTGGCCGGCCATCACTCCTCTTAGCAAA | This study |
| phcQA2C | TTTGCTAAGAGGAGTGATGG CCGGCCACCCCGCGGCGCCG | This study |
| phcQB2H | TCAAGCTTCCGGTTCGAGATGGTCAAGG | This study |
| phcA-C-FLAG | CTGGATCCTCACTTATCGTCGTCATCCTTGTAATCGATCTTATC  GTCGTCATCCTTGTAATCTCCCTTATCGTCGTCATCCTTGTAATC | This study |
| phcA-N-FLAG-A1 | GAGCCAAAGCGTGGATTACAAGGATGACGACGATAAGGGAGATTACAAGGATGACGACGATAAGATCGATTACAAGGATGACGACGATCCCATAAGGTCAACGTCGATACCAAG | This study |
| phcA-N-FLAG-B1 | CTTGGTATCGACGTTGACCTTATCGTCGTCATCCTTGTAATCGATCTTATCGTCGTCATCCTTGTAATCTCCCTTATCGTCGTCATCCTTGTAATCCATGGGACGCTTTGGCTC | This study |
| phcAdC30 | CTGGATCCTCACATCGGCGCGATGGAGG | This study |
| phcAdC10 | CTGGATCCTCACCGGTAAGGGCGATGCA | This study |
| clpBA1 | TGTCGTACATCGAAGTGATG | This study |
| clpBB1C | GTTCAAGGAGGCGTCGCGGCGAAAAAACCCCTTGCGGAAA | This study |
| clpBA2C | TTTCCGCAAGGGGTTTTTTCGCCGCGACGCCTCCTTGAAC | This study |
| clpBB2 | AACACGATGGTCGAGGTGTA | This study |
| clpAA1 | CGAAGCTTCCACAGCATGTCAAGGCTGC | This study |
| clpAB1C | GCTTGGGAACCACCGAACCGGCTTCCTCCATCACGCACTG | This study |
| clpAA2C | CAGTGCGTGATGGAGGAAGCCGGTTCGGTGGTTCCCAAGC | This study |
| clpAB2 | CTGGATCCACTCGACGTCCAGATCCTCG | This study |
| hlsUVA1 | CTGGATCCAACAAGCGCATGGAACAGTA | This study |
| hlsUVB1C | AGGACCCAAGGAACAAGCGCGCCCCGCGCCGCACAACCGC | This study |
| hlsUVA2C | GCGGTTGTGCGGCGCGGGGCGCGCTTGTTCCTTGGGTCCT | This study |
| hlsUVB2 | CGAAGCTTCAGCTTCTTGCGGAAGACCT | This study |
| lonA1 | GCGGATCCGGAAGACCTGATCAAGTT | This study |
| lonB1C | CCGGAAAGCGCATCACCTCATATTTTCCCCAGAAAGTCAT | This study |
| lonA2C | ATGACTTTCTGGGGAAAATATGAGGTGATGCGCTTTCCGG | This study |
| lon-B2 | CGAAGCTTGCTCTACCAACTGAGCTAAG | This study |
| rsc3101A1 | AAGGTGTTCGTCGAAGACAT | This study |
| rsc3101B1C | GGCGCGTGACGGCAAAACGCCACACTGGCTGAAAATACGC | This study |
| rsc3101A2C | GCGTATTTTCAGCCAGTGTGGCGTTTTGCCGTCACGCGCC | This study |
| rsc3101B2 | TGGATATCCACTACGTCGAT | This study |
| rsc1749A1 | AAGGGAATCGTCAGTCTGGA | This study |
| rsc1749B1C | GGCGGTTTCTGTCCTGCGCGCAGGGCCATG GTGTCTCCTC | This study |
| rsc1749A2C | GAGGAGACACCATGGCCCTGCGCGCAGGACAGAAACCGCC | This study |
| rsc1749B2 | GATGTAGTGGATGCCCTGGT | This study |
| rsp0650A1 | ACGGCTACTTCGTCTACGTC | This study |
| rsp0650B1C | AGAGGTCTCCGCATGGTGGCGGTATGTCTCCTGTGCAAGT | This study |
| rsp0650A2C | ACTTGCACAGGAGACATACCGCCACCATGCGGAGACCTCT | This study |
| rsp0650B2 | CCAGGTTGTTGCAGATCTTC | This study |
| rsp1552A1 | CGGTATCGATCGATACGGGT | This study |
| rsp1552B1C | TGCGCGCGGC TTGTGCTCCGGGTGGAAAACGAGATCCCGC | This study |
| rsp1552A2C | GCGGGATCTCGTTTTCCACCCGGAGCACAAGCCGCGCGCA | This study |
| rsp1552B2 | AGCATCAGGACAACCGACCG | This study |
| phcA-NLUC-BS-F | GGACGAGCTCGGTACCCATGGTCAACGTCGATACCAA | This study |
| phcA-NLUC-BS-R | TACGAGATCTGGTCGACGACGGACAGCCGCGACTCAT | This study |
| phcQ-CLUC-BS-F | CGTCCCGGGGCGGTACCATGACCAACCCCGGGGACAA | This study |
| phcQ-CLUC-BS-R | AAGCTCTGCAGGTCGAC TCAGGCGGCCTGCTGCTGCT | This study |
| phcA-pGEX4T-1-BS-F | GGTGGAAGTGGTGGATCCATGGTCAACGTCGATACCAAGCT | This study |
| phcA-pGEX4T-1-BS-R | TCCAGATCCACCGTCGACGACGGACAGCCGCGACTCA | This study |
| PhcAdC30-pGEX4T-1-BS-R | TCCAGATCCACCGTCGACCATCGGCGCGATGGAGGCCT | This study |
| phcQ-pET32a-BS-F | GGTGGAAGTGGTGGATCCATGACCAACCCCGGGGACA | This study |
| phcQ-pET32a-BS-R | AGCAGCAGCAGGCCGCCGTCGACGGTGGATCTGGA | This study |
| glmsdown | GCGCTCAAGCTCAAGGAGATC | Zhang *et al*., 2011 |
| Tn7R | CACAGCATAACTGGACTGATTTC | Choi *et al*., 2005 |

Choi, K.H., Gaynor, J.B., White, K.G., Lopez, C., Bosio, C.M., Karkhoff-Chweizer, R.R. and Schweizer, H.P. (2005) A Tn*7*-based broad range bacterial cloning and expression system. *Nat Methods.* 2, 443–448.

Zhang, Y., Kiba, A., Hikichi, Y. and Ohnishi, K. (2011) *prhKLM* genes of *Ralstonia solanacearum* encode novel activators of *hrp* regulon and are required for pathogenesis in tomato. *FEMS Microbiol. Lett.* 317, 75–82.
